# Current and Future Market Opportunities for Alternative Proteins in Low- and Middle-Income Countries

**DOI:** 10.1016/j.cdnut.2023.102035

**Published:** 2024-02-01

**Authors:** Resham Talwar, Mathilda Freymond, Kalpana Beesabathuni, Srujith Lingala

**Affiliations:** 1 Department of Biomedical Engineering, Johns Hopkins University, Baltimore, MD, United States; 2 Sight and Life, Basel, Switzerland

**Keywords:** alternative protein, investment, nutrition, sustainability, scalability, affordability, research and development, market opportunity, consumer behavior, demand

## Abstract

There is an urgent need for sustainable food systems to address the nutritional requirements of today and tomorrow. Alternative proteins (AP) have the potential to diversify the protein sources available for consumption while aligning with nutritional, environmental, and cultural needs and preferences. Although AP startups and investors focus on high-income countries, there is a growing market opportunity for AP in low- and middle-income countries (LMICs) due to increasing incomes, urbanization, and market expansion. This study aimed to evaluate the market opportunity for AP in LMICs by evaluating current global AP market trends, the factors influencing consumer demand, and the key aspects for enabling the environment for AP. Risks, challenges, and strategies for AP market expansion in LMICs are also discussed. The expansion and adoption of AP in LMICs could present a promising solution to nourish the world’s growing population while mitigating the global food and environmental crises.

## Introduction

The global population will reach ∼9 billion by 2050 [1]. Economic development and increased urbanization in low- and middle-income countries (LMICs) are expected to bring changes in lifestyles and consumption patterns, especially, increased consumption of protein-dense animal-sourced foods [2]. As the demand for meat rises, so does the concern over its negative environmental impact [2]. Alternative proteins (AP), including any protein-rich ingredients sourced from plants, insects, fungi, algae, or animal cells, present an opportunity for the consumption of protein-dense foods with a lower environmental impact than conventional livestock products [3].

The broad AP category of plant-based proteins, such as legumes and pulses, as well as insects and algae, present sensible solutions because they are traditional and local protein sources for many LMICs [4]. Although the AP industry has primarily focused on high-income countries (HICs) in the past few decades, there is a growing recognition that LMICs markets offer potential for market growth, driven by factors such as increasing disposable incomes, urbanization, and consumer awareness of the ethical and environmental implications of livestock production [5].

Similar to HICs, the demand for AP in LMICs will vary according to social, cultural, economic, and environmental factors. This perspective aimed to shed light on the market opportunity for AP in LMICs by uncovering the current global AP market trends and size and examining the factors influencing consumer demand. Additionally, it proposes multi-level strategies that can help enable the environment for AP and discusses challenges and opportunities for market expansion in LMICs.

## Alternative Protein Market Size and Trends

The global AP market is growing across regions. The Asia-Pacific region accounts for the fastest-growing AP market, with an expected compound annual growth rate (CAGR) of 18.5% from 2021 to 2027 [6,7]. The CAGR helps determine the growth and rate of return in an industry or market. The rise of AP in the Asia-Pacific region, and others globally, is driven by several factors such as cultural and religious considerations, the growing health consciousness, and the increasing consumption of healthy food ingredients [6]. The growth of the industry can also be influenced by the availability of raw materials and low-wage labor in countries such as China and India [7].

In the United States, the AP market exceeded 50 billion USD in 2020 and is estimated to grow at >17.5% CAGR between 2021 and 2027 [6]. Plant-based AP surpassed 35 billion USD in 2020 and is projected to keep growing, as are other AP [6]. In the United States, soy, wheat, and peas are currently among the most consumed plant-based protein sources that offer various applications in the food and beverage industry, ranging from meat and sausage alternatives to high-protein dairy alternatives [7]. In Europe, the plant-based protein market is growing with a CAGR of 12.0% between 2021 and 2028 and is expected to reach 7 billion USD by 2028 [8]. Other regions with an expanding plant-based protein market are the Middle East and Africa where the trend goes up to a CAGR of 10.3% and the market is expected to reach 1.1 billion USD by 2028 [8].

Although the AP market is projected to grow globally, its entry and expansion in LMICs encounter obstacles due to various factors, including cultural barriers, regulations, education, affordability, and scalability of the supply chain [9]. The following section describes the most relevant determinants for AP consumption, in an effort to shed light on the market opportunities and challenges.

## Factors Influencing Consumer Demand for Alternative Protein: Consumption Drivers

### Social, cultural, and environmental factors

Many social and cultural factors can affect the demand for AP. Despite growing awareness of the downsides of high animal protein consumption in HICs, meat consumption habits and limited availability of plant-based products hinder the adoption of a more plant-based diet [10].

AP products should be adapted to local flavors and textures, allowing integration into existing diets and culinary traditions [11]. For example, GoodDot, an Indian AP company specializing in meat alternatives made from soy, pea, and other plant-based protein sources, has a demand generation strategy focused on taste and familiarity with products mimicking popular Indian dishes, such as kebabs and chicken curries [12]. These aspects will be essential for the transition to plant-based products that are more appealing to LMICs consumers.

Environmental and sustainability factors have also become major drivers for the transition to a plant-based diet in HICs. However, this is not yet the case in LMICs; consumers prioritize nutritional value, food preferences, sociocultural factors such as traditions and habits, and market food prices [13]. As consumers in both HICs and LMICs become more aware of the sustainability and animal welfare issues associated with conventional meat production in the long term, they may be more likely to consider AP options [14].

### Economic factors

#### Consumers’ price sensitivity

AP products are currently priced at a significant premium. To compete with traditional products in LMICs, they must become more affordable [15]. For example, consumers in HICs ranked price as the second-most important factor (behind taste) to motivate or restrict purchasing a plant-based product. In LMICs, affordability is often mentioned as a key factor influencing purchasing decisions [13]. On average, in HICs, plant-based meat is twice as expensive as beef, > 3 times as expensive as pork, and > 4 times as expensive as chicken per kilogram [15]. In the United States, the most affordable plant-based meat options are offered at prices roughly comparable to organic, grass-fed conventional ground beef. This means that plant-based meats are currently being bought by customers in the same socioeconomic groups as those who purchase organic, grass-fed conventional beef [15]. This will represent a challenge for the LMICs, where the purchasing power is even lower than in the HICs.

A recent online survey in Africa published in 2022 studied the potential adoption of plant-based meat, including current familiarity, acceptance, motivations, and desired product characteristics among nationally representative adults, including the Gen Z and Millennial age groups (18–39 y). About half of participants lived in a large city, 25% in a middle-sized town, and the rest in small towns or rural areas. The survey reported that after being introduced virtually to plant-based meat products, some consumers in African countries were willing to pay more for these compared with conventional meat. For instance, 50% of respondents in Kenya reported a willingness to pay more for those products, followed by Nigeria (46%), and Egypt (31%). Meanwhile, at price parity, an additional 24% in Kenya, 32% in Nigeria, and 20% in Egypt were willing to purchase plant-based meat products [16].

Understanding how consumers prioritize price and perceive the value of plant-based products can help inform industry decisions, thereby supporting investment, marketing campaigns, and product pricing.

### Economies of scale

Increased scale of production and economies of scale will be essential to help close the price gap. This has already been shown in HICs across categories such as milk and butter, where consumers pay a premium of 11% and 7%, respectively [15]. This price premium is lower than that for meat alternatives to beef or chicken, where consumers pay premiums of 105% and 324%, respectively [15]. The progress on price parity will not only be achieved by reducing the production costs of AP products but also by market effects, such as higher input costs, higher worker wage rates, supply chain interruptions, and animal welfare policies, which might raise the costs of conventional meat [15].

### Economic opportunities

Considering a diverse range of local AP will help promote sustainable and equitable development. Besides creating additional job opportunities for women and youth, the expansion of local AP industries will bring new opportunities for smallholder farmers [17]. Compared with industrial production in HICs, smallholder farmers play a key role in providing necessary proteins in emerging economies [18].

The emergence of AP alongside traditional animal-source proteins could offer broader choices for farmers regarding the production processes and greater resilience [17]. Additionally, the development of the AP industry in LMICs could allow for investment in new infrastructure and the development of local supply chains [19]. This can be done through partnering with local groups that specialize in areas like food sustainability, healthy eating habits, and nutritional needs of the society, thus supporting emerging startups and companies and utilizing innovative policy measures [20,21].

### Enabling environment for alternative protein

#### Investments

Investments are required to advance the AP market across different regions. The AP sector received a considerable amount of investments, with 11.2 billion USD invested over the past 3 y [22]. However, these investments have focused mostly on HICs. Investments in LMICs are needed to support the development of safe and nutritious AP products, market research to adapt AP to local needs, and entrepreneurial support to expand the local sourcing of ingredients [18]. Few LMICs are already observing some transitions in government investment. For instance, India has the world’s first cellular agriculture research center. Moreover, the Ministry of Agriculture and Rural Affairs in China has included the research and development of plant-based eggs and cultivated meat in its 5-y plan [23]. These milestones suggest that investment is a priority in creating space for AP in the LMICs markets.

#### Scalability and supply

Besides increasing investments, AP companies must be prepared to scale up production to achieve commercial viability [20]. The scalability of AP in LMICs is essential to reach price parity and become affordable. At this point, a lack of infrastructure and weak supply chains pose challenges for scaling local AP production [24].

Quorn, a United Kingdom–based meat-substitute company utilizing the fungal species *Fusarium venenatum* to create mycoprotein, was acquired by Monde Nissin in 2015 to enter the Asia-Pacific market [25]. Monde Nissin’s deep knowledge of the commercial and local nuances helped Quorn enter the Thai market successfully [25]. Fermentation is a resource-efficient and highly scalable food processing process. According to Quorn, fermentation with ∼1 g of *Fusarium venenatum* can create >1500 tons of mycoprotein [25]. With this efficiency, the company can afford to sell its products at a very competitive price. For example, in Southeast Asia, Quorn burger patties are 4 times less costly than the plant-based Beyond Burger patties, costing 7 SGD for 4 patties compared with ∼14 SGD for 2 [25]. Local sourcing and community support in the supply chain increased the accessibility and affordability of these products for consumers. Quorn's scalability and strategic pivot into the Asia-Pacific market serve as a successful example of how to scale up AP in other regions.

International restaurant chains can play a significant role in increasing the sales, accessibility, and demand consumption of AP [26]. Additionally, conventional meat companies are also transitioning to supply AP. Examples include Tyson, JBS Foods, Unilever, Nestlé, and Smithfield, all of which invest in the AP sector to expedite research and development and expand their portfolio with diverse protein sources [27]. For instance, Unilever invested <85 million Euros in a foods innovation center at Wageningen University in the Netherlands to support research on plant-based ingredients and meat alternatives, efficient crops, sustainable food packaging, and nutritious food [28]. Barriers for new local companies in LMICs include logistical challenges with a certain lack of infrastructure, such as cold storage and transportation, while also facing competition from well-established animal protein firms [29].

#### Market competition

Increasing competition in the market can lead to more options for consumers, decreased costs, and increased acceptance of APs [29]. As the AP market grows, competition between companies could also lead to market saturation, i.e., market growth trajectory stagnation and negative economic implications. A saturated market limits a company’s profitability and growth potential [30]. Therefore the market growth in HICs and LMICs needs to be diversified and scalable, which will allow such stagnation and allow companies to tap into new markets with their niche and adapt to such market challenges [31].

### Strategies for Market Expansion

There are different strategies for market expansion at the global, national, industry, and individual levels (Table 1) [32]. Examples of this include large-scale opportunities for the market expansion of AP through collaborative efforts across borders through partnerships between governments, international organizations, and private companies to support the development of AP in LMICs. These strategies include engaging with stakeholders, local sourcing, and branding, among others. Companies manufacturing AP can reach a large market using existing automation and technology capabilities, rethinking marketing approaches, and driving the adoption of these novel foods by educating and building robust production and supply networks [20].

**TABLE 1:**
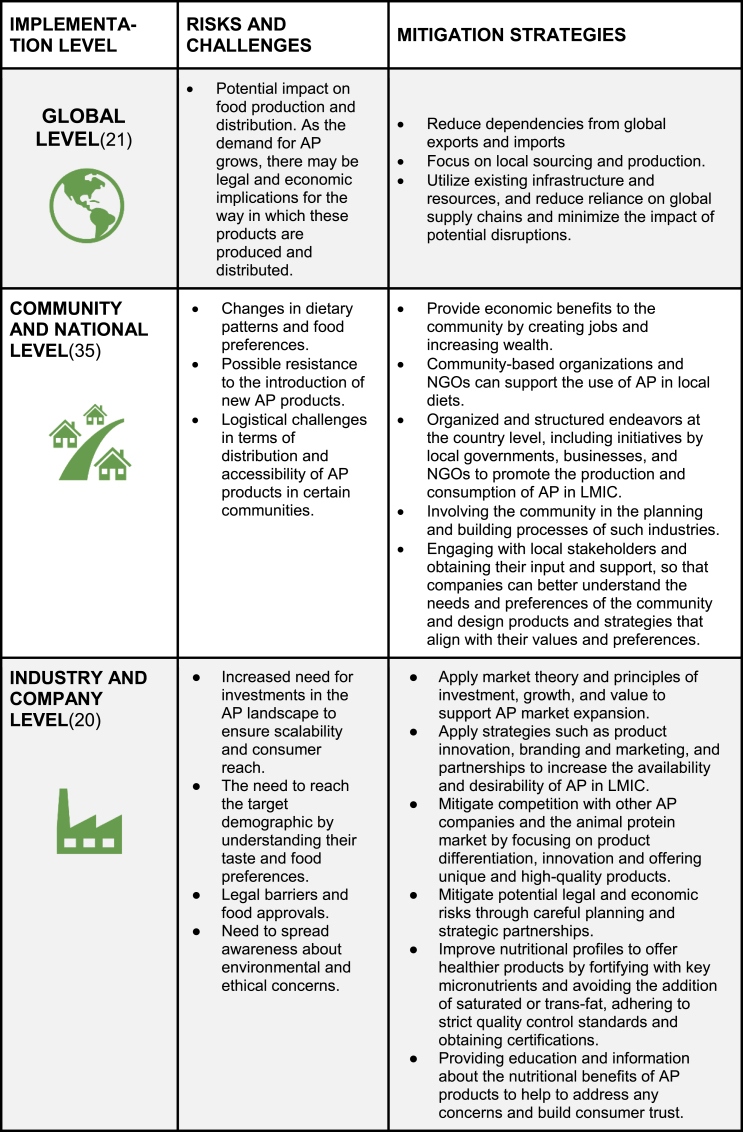
Risks, challenges, and strategies for alternative protein market expansion at different implementation levels

*Abbreviations:* AP, alternative proteins; LMIC, low- to middle-income country; NGO, nongovernmental organization.

## Continued Research and Collaboration

Besides the need for more development and research within the AP industry, more research on the impacts that AP may have on areas such as nutrition, the environment, and livelihoods is essential [33]. These impacts should be modeled and measured as they manifest and AP scale up [33]. The impacts are expected to be heterogeneously distributed, and focus should be placed on the differences across demographic groups, the magnitude of impacts, and the timescale over which these impacts appear [33]. Partnerships with local organizations and governments, as well as funding and investments, can facilitate research. Additionally, impact evaluation will help identify factors that facilitate and constrain the transition to APs [33].

Global coalitions such as The Protein Challenge 2040 bring together the animal, plant, and novel protein industries, as well as the global environmental and health organizations [34]. This partnership helps these industries take action to shift into more sustainable production and consumption of protein. They have identified the following 3 areas for immediate action: *1*) increasing the proportion of plant-based protein consumption, *2*) scaling up sustainable animal feed innovation to meet the demand for animal protein, and *3*) closing the protein nutrient gap [34]. The initiative promotes environmental sustainability and nutritional value to drive consumer demand. It has also invested in and supported local farmers and small-scale AP businesses to improve the availability of these products in the market. Behind The Protein Challenge 2040 is a large consortium of organizations including nongovernmental organizations (NGOs) such as the World Wildlife Fund and the Global Alliance for Improved Nutrition, the nutrition and flavor expert DSM Firmenich, and companies and retailers such as Quorn, Target, and Waitrose, among others [34]. Collaboration between governments, businesses, and NGOs promises ways to increase awareness and accelerate progress toward the sustainable production and consumption of protein.

## Conclusion

As the world population continues to grow, ensuring adequate, nutritious, and sustainably sourced food for all remains a priority. The AP industry has a crucial role to play in meeting this demand, and with continued research, innovation, and investment, it can help shape a more hopeful and sustainable future for all. Economic development and increased urbanization in emerging economies are expected to bring a higher consumption of high-quality animal protein. As a result, these countries are at the forefront of the expanding AP market and are well-positioned to benefit from it. To determine the feasibility of adopting specific AP products, it is crucial to evaluate the market opportunities in each region.

For successful establishment and operations in LMICs, AP companies must consider a range of components influencing consumer demand, such as social, cultural, environmental, and economic factors. The 2 key aspects include offering AP products that are familiar in taste and texture and ensuring product affordability. These factors will increase the demand for AP products and increase dietary diversity. Furthermore, using local sources of diverse proteins within the LMICs markets will improve the sustainability and viability of the market by supporting the local economy and providing new job opportunities.

Expanding AP in LMICs presents a valuable opportunity for AP companies to meet the growing demand for nutrient-dense, sustainable, and ethically produced food products. The strongest drivers of market opportunity for AP will likely include price, attitudes toward health and nutrition, and increasing awareness of the environmental and ethical impacts of conventional animal-sourced protein production.

The expansion of AP could provide nutritious solutions while also diversifying the protein-rich food basket and addressing concerns about environmental impact, animal welfare, and human health. With continued investment in research and infrastructure, collaboration, and dedication, access to quality, affordable, and sustainable sources of protein is possible for LMICs and globally. AP can contribute to a more nourished and equitable world, and this opportunity should be further capitalized on to create a better future for generations to come.

## Author contributions

The authors’ responsibilities are as follows – RT, MF, KB, and SL: conceptualized the outline for the paper; RT: conducted the literature review, wrote the first draft of the manuscript, wrote and structured the final draft, compiled Table 1, and added references; MF: supported with literature review, article structure, writing of subsequent drafts, and the references; SL and KB: provided inputs into the analysis and editing of the manuscript; SL: led the submission process; and all authors: read and approved the final manuscript.

## Conflicts of interest

The authors report no conflicts of interest and did not receive any funding from the commercial or private-sector entities for this research. RT was a voluntary participant in the Alternative Protein Project Chapter at Johns Hopkins University that collaborates with the Good Food Institute, but financial compensation was not received during this participation. This research did not involve human subjects; therefore, it was exempt from institutional review board requirements.

## Data Availability

Data described in the manuscript, code book, and analytic code will be made available upon request pending application and approval.
